# Variations in Movement and Habitat Selection by Eurasian Curlew at Two Important Sites in Europe Throughout Winter

**DOI:** 10.1002/ece3.74164

**Published:** 2026-08-24

**Authors:** Marie Donnez, Philipp Schwemmer, Moritz Mercker, Stefan Garthe, Frédéric Jiguet, Andreas Bange, Loïc Jomat, Pierre Rousseau, Pierrick Bocher

**Affiliations:** ^1^ Laboratoire Littoral Environnement Et Sociétés, UMR LIENSs 7266 CNRS‐La Rochelle University La Rochelle France; ^2^ Research and Technology Centre (FTZ) University of Kiel Büsum Germany; ^3^ Bionum GmbH – Consultants in Biological Statistics Hamburg Germany; ^4^ CESCO, UMR7204 MNHN‐CNRS‐Sorbonne Université Paris France; ^5^ National Nature Reserve of Möeze‐Oléron Ligue Pour La Protection Des Oiseaux (LPO), La Grange à Noureau Saint‐Froult France

**Keywords:** coastal ecology, GPS tracking, home range, *Numenius arquata*, rhythm activity, waders, wintering season

## Abstract

Shorebirds aim to replenish their body reserves during the wintering season to ensure their subsequent survival and to improve their breeding performance. However, their space use depends on their fluctuating resource availability and energy requirements throughout the winter. Hence, this study aimed to determine if the space and habitats used by Eurasian curlews ( *Numenius arquata* ) were influenced by their wintering site and stage. Sixty‐two curlews were equipped with GPS tags at two major wintering sites in Europe with contrasting waterbird density: the Pertuis Charentais (PC) in France and the German part of the Wadden Sea (WS). Their home ranges (HRs) and foraging habitat selection were compared according to three wintering stages requiring different energy demands (i.e., early winter, mid‐winter and late winter before migration). Curlews exhibited similar HR sizes at both sites throughout the winter, suggesting no effect of waterbird density on individuals' space use. HR sizes were larger during the day than at night and showed high inter‐individual variability, unrelated to sex. Notably, the HRs were larger during the first winter stage (i.e., Arrival–September), with curlews spending a greater proportion of time on mudflats, and selecting distinct foraging areas located further from the coast at this stage. Curlews in the WS fed mainly on the mudflats, while curlews in the PC fed on the mudflats, but also on biogenic reefs, oyster parks and seagrass beds, the latter being favoured during the first stage. These results suggest the need for greater consideration of the distribution of curlews and other shorebirds at the beginning of the wintering season to define areas and habitats of importance to support future management and conservation measures. Indeed, birds are particularly prone to disturbance during the first winter stage, when tourism pressure on the coast is high and birds tend to be more mobile.

## Graphical abstract

This study aimed to determine if the space and habitats used by Eurasian curlews ( *Numenius arquata* ) were influenced by their wintering site and stage. A total of 62 curlews were equipped with GPS‐GSM tags at two major wintering sites in Europe and their home ranges and foraging habitat selection were compared according to three wintering stages requiring different energy demands (i.e., early winter, mid‐winter and late winter before migration).

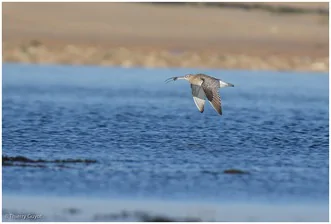

## 1 Introduction

Shorebirds must choose wintering sites that provide all the essential resources required for their feeding and resting activities within an accessible portion of the local landscape (Dunning et al. 1992; Savary et al. 2024), primarily using habitats that optimally fulfil these two biological functions (Hildén 1965; Doligez and Boulinier 2018). When levels of intra‐ and interspecific competition at feeding areas increase, however, and prey availability decreases during the winter, shorebirds are likely to rely on other feeding areas by emigrating to a new habitat (Morris 1992; Sutherland 1996). Although some individuals will be able to use multiple suitable habitats and functional sites (e.g., black‐tailed godwits *Limosa limosa* ; Gill et al. 2007, Jourdan et al. 2022), some individuals will be forced to supplement their resources in lower quality habitats (Brown 1969; Gill et al. 2001), potentially affecting their survival and fitness (Hildén 1965; Orians and Wittenberber 1991).

During the wintering season, shorebirds have also to deal with different energy‐demanding events according to the stage of the winter, such as primary moulting (Remisiewicz 2011), predation risks (van den Hout et al. 2008; Dekker and Drever 2016), and severe weather, e.g., low temperatures and high winds that increase the energetic costs of thermoregulation (Davidson 1981; van der Kolk et al. 2020). To ensure their survival and improve their subsequent breeding performance (Zwarts et al. 1990; Morrison and Hobson 2004), it is thus crucial that they replenish their body reserves by selecting habitats that maximise their net energy intake rate throughout the winter (van Gils et al. 2003).

Among shorebird communities, the Eurasian curlew *Numenius arquata* (hereafter curlew) is able to use a wide diversity of coastal and inland habitats during its wintering season (van de Kam et al. 2004; Donnez et al. 2023). On the western coasts of Europe (Delany et al. 2009), curlews regularly reach their wintering site in early summer (early June to late July) and leave in early spring (early March to late April) to reach their breeding grounds from northern Europe to the Arctic Circle (Amélineau et al. 2021; Pederson et al. 2022; Donnez et al. 2023). They feed primarily on intertidal mudflats during the wintering season (Lack 1986; Colwell 2010), preferentially on polychaete worms and crabs, according to the tidal rhythm (Goss‐Custard et al. 1977; Schwemmer et al. 2012; Bocher et al. 2014). Although shorebirds commonly aggregate in dense, mixed‐species flocks using the same feeding areas (Metcalfe 1989; Cestari et al. 2020), curlews tend to exhibit territorial behaviour both during the breeding (Currie and Valkama 2000) and wintering seasons (Ens 1979; Colwell 2000; Summers 2019). At high tide, curlews generally aggregate at nearby supratidal roosts in groups of several hundreds or thousands of individuals (Brown 2015), where they can stand on wet or dry substrates (Rogers et al. 2006; Rosa et al. 2006). The species has undergone rapid population declines across Europe, with an estimated decreasing by 30%–49% in 31.2 years (BirdLife International 2015), making the species ‘Near Threatened’ on a global scale. To counter that trend, habitats selected by curlews throughout the winter must be defined in order to allow the rapid and efficient implementation of strategic habitat management decisions and thus improve curlews' winter survival.

Hence, this study aimed to compare space use and habitat selection by curlews according to the distinct characteristics of their wintering sites, from their arrival at the sites to their departure towards the same breeding areas in north‐eastern Europe (Bocher et al. 2024), to detect potential differences in wintering strategies between (1) the populations and (2) the different winter stages. To this end, we deployed GPS tags on 62 wintering curlews within two major wintering sites in Europe: the Pertuis Charentais (PC) in France and the German part of the Wadden Sea (WS). Although both sites are dominated by large intertidal mudflats, they differ in terms of the number of wintering and staging waterbirds, availability of intertidal areas, landscape structures and regulation of human activities.

We hypothesise that curlews in the WS would use more space (Donnez et al. 2023) and exhibit a longer foraging time than those in the PC as a result of the higher density of waterbirds in the WS, potentially forcing curlews to cover more space to reduce the level of competition for resources (Leone and Estévez 2008). Whatever the site, we assume that males and females have the same HR size throughout the winter (Mander et al. 2022), and that individual HRs are larger during the day than at night because of difficulties in detecting prey visually at night (Turpie and Hockey 1993). In particular, we hypothesise that curlews' home ranges would be larger in WS during late summer and autumn, when curlews arrive at their wintering site and large numbers of waterbirds stop in this site to replenish their body reserves before continuing their migration (Meltofte et al. 1994), and that curlews would use the same foraging areas throughout the wintering season, preferentially located on mudflats (Lack 1986; Colwell 2010), due to their high fidelity to their wintering site across the years (Schwemmer et al. 2016, 2026; Sanders and Rees 2018).

## 2 Methods

### 2.1 Study Sites

**FIGURE 1:**
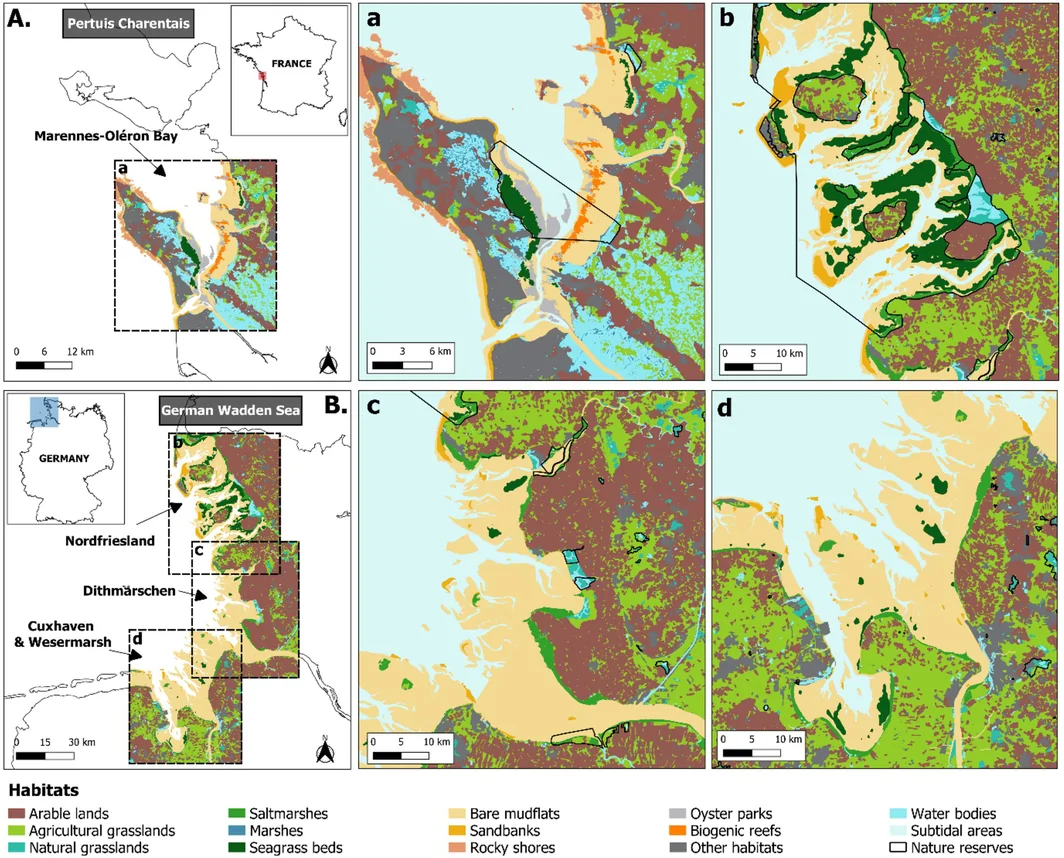
Habitat mapping of (A) the Pertuis Charentais and (B) the German Wadden Sea and localization of the study areas: (a) Marennes‐Oléron Bay and coasts of (b) Nordfriesland, (c) Dithmarschen, and (d) Cuxhaven and Wesermarsch districts.

The analyses were carried out at two major wintering sites for curlews in Europe, located on the East‐Atlantic Flyway: the PC (46°02′ N, 1°52′ W) on the French Atlantic coast and the German part of the WS (53°31′ N, 8°33′ E) (Figure 1).

The coast of the PC consists of a series of straits bounded by islands and mainland and dominated by particularly rich and productive large intertidal bare mudflats (Verger 2005, Figure 2), recognised to be of international importance for waterbirds (Delany et al. 2009). This is considered the most important wintering site for shorebirds in France (Mahéo et al. 2024), with about 150,000 waterbirds recorded each year (Wetlands International counts), including about 5760 curlews. All the analyses were restricted to the Marennes‐Oléron Bay (45°55′ N; 1°10′ W) (Figure 1a), which is located in the southern part of the PC and in the direct vicinity of the Charente and Seudre estuaries. It extends over 150,000 ha bordered to the west by the dune ridges of Oléron island and to the east by the Brouage mudflats. It is the second most important host site for curlews in France after Mont‐Saint‐Michel Bay, with about 2700 wintering individuals (Mahéo et al. 2024). Since 1985, part of the bay has been included in the National Nature Reserve of Moëze‐Oléron (approx. 6700 ha), which includes 5000 ha of mudflats and several adjacent marshes that provide important roosts for shorebirds.

**FIGURE 2:**
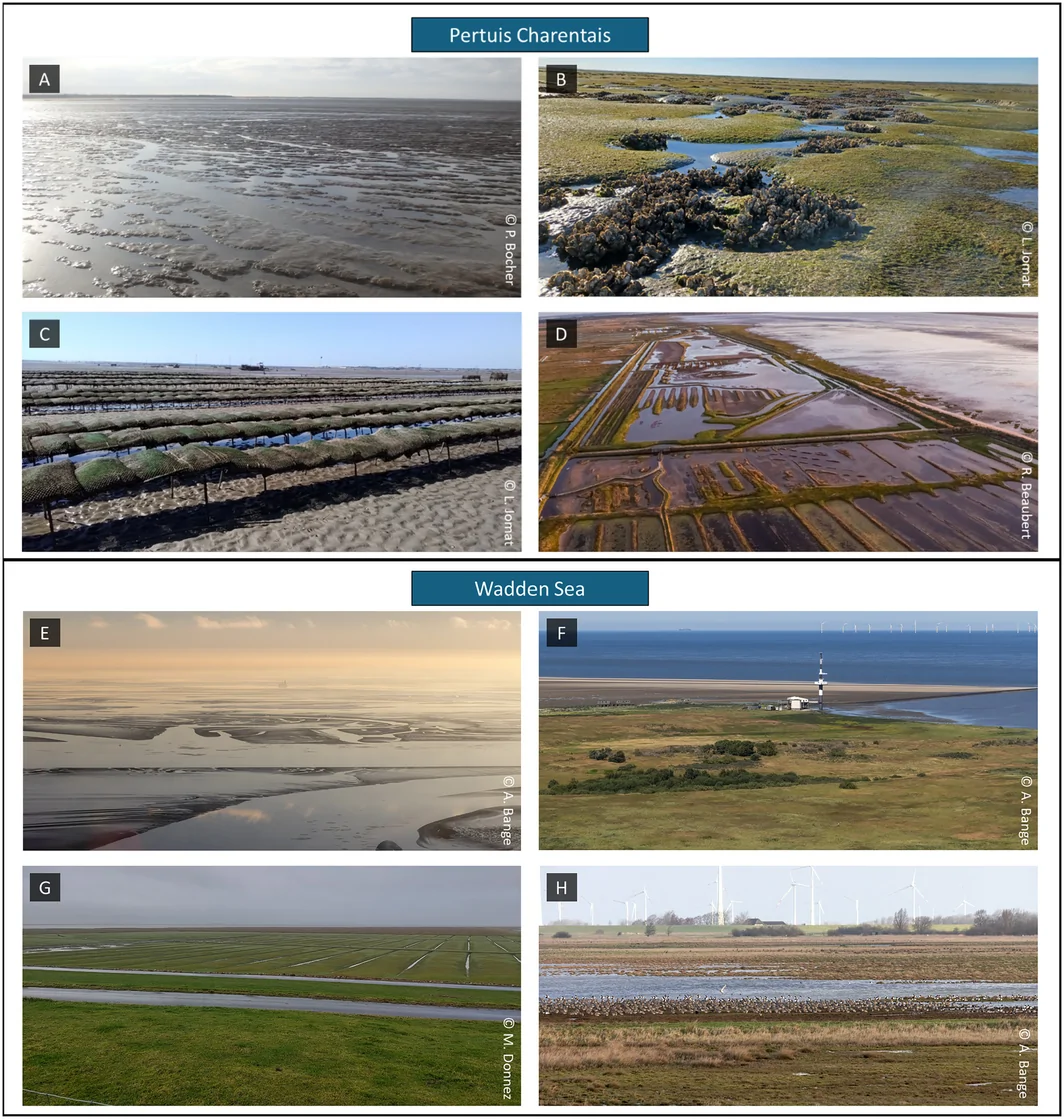
Main feeding (A–C, E) and roosting (D, F–H) habitats in the Pertuis Charentais (PC) and Wadden Sea (WS). (A) Bare mudflats, (B) seagrass beds and biogenic reefs, (C) oyster parks, (D) coastal marshes in the PC, (E) bare mudflats, (F) island, (G) saltmarshes, and (H) natural grasslands in the WS.

The WS is a large coastal wetland bordering the coasts of The Netherlands, Germany and Denmark. It contains the largest unbroken system of tidal flat areas and sandbars in the world, much of which was declared a UNESCO World Heritage Site in 2009 and 2014 (Figure 2). Around 10–12 million waterbirds use the WS in the course of a year (Meltofte et al. 1994), including about 250,000 curlews (Kleefstra et al. 2022), making the entire WS the most important wintering site for curlews in Europe (Wetlands International counts). Birds were studied along the German WS, which hosts approximately 80,000 curlews (Kleefstra et al. 2022), including the coasts of Nordfriesland (Figure 1b) and Dithmarschen (Figure 1c) districts in the federal state of Schleswig‐Holstein and the coasts of the Cuxhaven and Wesermarsh (Figure 1d) districts in Lower Saxony. Three national parks with a total area of 796,750 ha were established between 1985 and 1990 to limit the disturbance to birds caused by human activities, including most of the tidal flats and adjacent saltmarshes used by waterbirds as roosting and/or feeding habitats. In addition, nature conservation measures within recently embanked polders also help to maintain roosts for multiple bird species.

### 2.2 Habitat Description

The CORINE Land Cover (CLC) data from 2018 were used to similarly describe the habitats with a minimum area of 25 ha in France and 5 ha in Germany (Büttner 2014, Figure 1). These databases allowed the delineation of three coastal habitat categories: ‘bare mudflats’ (CLC code: 423) with estuaries and rivers also considered as mudflats at low tide (codes: 511, 522), ‘sandbanks’ (code: 331), and marshes (code: 421). Marshes were considered as ‘saltmarshes’ in the WS, i.e., marshes periodically flooded by seawater, and as ‘marshes’ in the PC, corresponding to basins into which seawater is artificially fed. We additionally employed site‐specific habitat layers to describe the typology of the coastal and intertidal habitats particularly used by curlews in more detail (see habitats described in Hilgerloh et al. 2001, Navedo and Masero 2008, Donnez et al. 2023). Hence, ‘seagrass beds’, ‘oyster parks’, ‘biogenic reefs’ resulting from former oyster parks and ‘rocky shores’ were delineated in the PC using a portable GPS for the first two habitats, and manually using satellite imagery for the last two habitats. Coastal and intertidal habitat layers of the WS were collected using Mendeley Data https://doi.org/10.17632/2rvtxpjtfg.1 (Baptist et al. 2019), allowing the delineation of ‘seagrass beds’ and to adjust the delineation of ‘saltmarshes’ and ‘sandbanks’ (i.e., coarse sand with a median grain size of 250–2000 μm and a silt percentage < 25%; Bouma et al. 2005).

### 2.3 Water Levels

Water levels in Marennes‐Oléron Bay (PC) were obtained from 2016 to 2024 on the Naval Hydrographic and Oceanographic Service portal (SHOM, https://www.shom.fr/, ‘Ile d'Aix’ station, mean (± standard deviation (SD)) distance to curlews = 13.6 ± 2.8 km). The dataset contained some gaps, and the tide signal was reconstructed using the Matlab ‘UTide’ package (Codiga 2011) with Python programming software (van Rossum and Drake 2009), to obtain the approximate water level at a regular time interval of 5 min. The approximate times of high and low tides were then extracted using R statistical software (version 4.4.1, R Core Team 2024). The times of high and low tides and 1‐min water level in the WS were derived from the Federal Waterways and Shipping Administration provided by the Federal Institute of Hydrology in Germany. The values were measured at six hydrographic stations (mean distance to curlews = 9.6 ± 4.4 km) considered to be the closest to the areas frequented by the tracked individuals.

### 2.4 Catching and Tagging of Curlews

A total of 62 adult wintering curlews were included in this study, comprising 35 birds caught in the Marennes‐Oléron Bay in the PC (France) between 2016 and 2023 and 27 on the coast of three distinct districts in the WS (Germany) between 2014 and 2023 (4 at Nordfriesland, 5 at Dithmarschen, 18 at Cuxhaven and Wesermarsch districts) (Figure 1, Table 1). All birds were caught directly at their wintering site using mist nets placed at high‐tide roosts during new‐moon nights. Each bird was ringed and weighed at the time of capture, and the bill, tarsus and wing length were measured to the nearest millimetre. The sex of each individual (30 females and 30 males) was estimated either morphologically using bill length (females have longer bills than males; Summers et al. 2013), or genetically using feather samples (Tauros Diagnostics, Berlin, Germany), with no difference in sex determination between the molecular and morphometric methods (Pederson et al. 2022). Two individuals could not be sexed. The birds were aged (53 adults and 9 juveniles) according to their plumage patterns (Demongin 2016). If birds had recorded data for multiple years, only the wintering season with the most locations was analysed. For curlews tagged as juveniles, data for the first wintering season after their first breeding attempt were retained, when the individuals were considered as adult birds. Among the retained birds, 27 had usable data for a complete wintering season, while 6 birds changed site during the winter or had less than 1000 GPS fixes during a winter stage, and no complete season was recorded for the other birds due to tag failure, loss of the device, or death of the bird; these birds were therefore only used for one or two of the three analysed stages (Table 1).

**TABLE 1:** Overview of tagging information and site description for curlews wintering in the entire Pertuis Charentais (PC) and Wadden Sea (WS).

	Tagged adults (*n*)	Males (*n*)	Females (*n*)	Unsexed (*n*)	Arr–Sep analyses (*n*)	Oct–Jan analyses (*n*)	Feb–Dep analyses (*n*)	Complete event (*n*)	Years of study	Intertidal area size (ha)	Punctual number of waterbirds	Density of waterbirds (ind./km^2^)	Number of curlews
PC	35	16	18	1	20	24	29	14	2016–2024	24,385^a^	150,000^c^	615	5760^e^
WS	27	14	12	1	22	20	19	13	2014–2024	537,541^b^	5 M^d^	930	250,000^f^
Total	62	30	30	2	42	44	48	27					

*Note:* A different number of birds was used per winter stage (Arr–Sep: Arrival–September, Oct–Jan: October–January, Feb–Dep: February–Departure). The number of birds used throughout the three winter stages is shown in the column ‘Complete event’. Density of waterbirds estimated according to intertidal area size.

^a^ Bocher et al. (2013).

^b^ Baptist et al. (2019).

^c^ International Wetlands Counts.

^d^ van Roomen (2024).

^e^ Mahéo et al. (2024).

^f^ Kleefstra et al. (2022).

The birds were equipped with solar GPS‐Global System for Mobile Communication tags provided by two manufacturers: the 10 birds captured before 2018 were equipped with Ecotone tags (Poland; Skua, 17 g, *n* = 8, or Sterna, 7.5 g, *n* = 2) and the 52 birds captured after 2018 were equipped with Ornitela tags (Lithuania; OT‐10, 10 g, *n* = 1, OT‐15, 15 g, *n* = 17, OT‐20, 20 g, *n* = 1, or OT‐E10, 12 g, *n* = 33). None of the tags exceeded the recommended 3% of the bird's body mass (Phillips et al. 2003). Tags were fitted in France using the ‘leg‐loop’ method (Mallory and Gilbert 2008) with harnesses made of silicone analytical tubes (*n* = 35), while tags deployed in Germany were fitted using the ‘wing‐loop’ method (Guillaumet et al. 2011) with Teflon harnesses (*n* = 27). Both attachment methods are known to induce contrasting effects on birds' survival (Thaxter et al. 2016), tracking duration (Weiser et al. 2025), flight performance (Longarini et al. 2023; Katzner and Young 2024), energy expenditure (Mizrahy‐Rewald et al. 2023) and comfort (Mallory and Gilbert 2008), potentially instigating site‐biased behaviour of individuals. However, these adverse effects are particularly pronounced during migration, and we therefore assumed no significant impact on our results and conclusions. Depending on the GPS device and the battery charge, which was highly influenced by weather conditions, GPS fixes were collected at intervals of 1–3843 min (98% at 1–60 min) over the monitored stages.

### 2.5 Feeding vs. Resting Positions

The approximate start of the wintering season was defined when the bird reached one of the two study sites and stayed there > 20 days. Then, the position of the first wintering roost used at the beginning of the season was determined by isolating the GPS fixes obtained during the first 3 days of the wintering season for each individual and displaying them on a 0.0001° × 0.0001° grid covering the range of latitudes and longitudes visited by each individual during these days. The centre of the cell with the largest number of GPS fixes was defined as the first roost (see method for nest‐position extraction in Amélineau et al. 2021 and Bocher et al. 2024). The corrected start and end of the wintering season were defined as the times when birds arrived at < 20 km from their first wintering roost and moved > 20 km away for the first time to start their spring migration, respectively (Pederson et al. 2022; Donnez et al. 2023).

**FIGURE A1:**
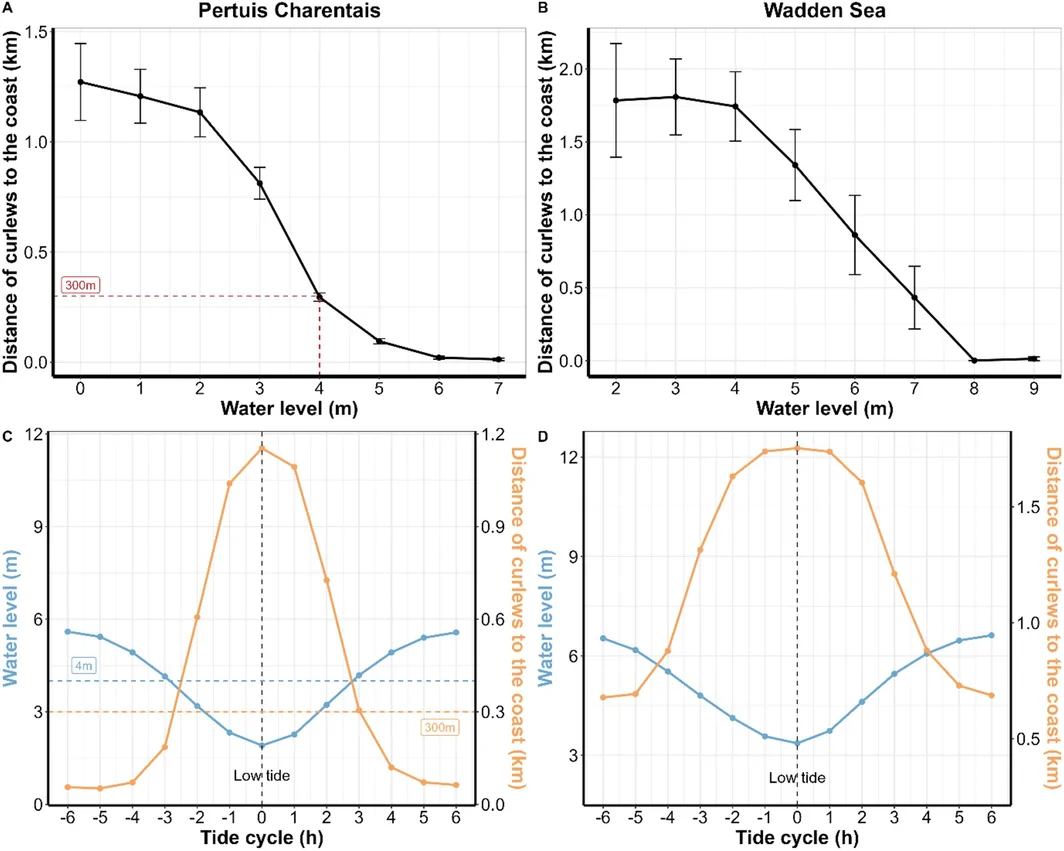
Mean (± standard error) distance of curlews to the coast according to the water level (A, B) and distance to the coast and water level according to the tidal cycle (C, D) in the Pertuis Charentais (PC: left column) and the Wadden Sea (WS; right column). Some curlews wintering in the PC rested in the upper part of the mudflats, up to about 300 m off the coast. We examined the corresponding mean water level (i.e., 4 m, A) and approximate time of the tidal cycle (i.e., ±3 h, C). It was not possible to apply the same methodology in the WS because curlews can stay longer on open parts of the mudflats (B, D).

GPS fixes were then separated into foraging (i.e., when birds were foraging for 3 h before and after low tide) and resting categories (i.e., when birds were resting 3 h before and after high tide). This 3‐h threshold was chosen by plotting the GPS fixes on a map and visually defining the distance to the coast at which PC curlews were most likely to feed (i.e., 300 m), preroosts position being known, and determining the average water level at which curlews were located at this distance in the PC (i.e., 4 m). It was then defined by the average time of the tide cycle when the average water level was 4 m (i.e., about 3 h before and after low tide, see Figure A1). This threshold was consistent with the estimated foraging time of bar‐tailed godwits in this site (Jourdan et al. 2021b). As WS curlews could stay on mudflat during high tide due to the presence of shoals, the same method could not be applied, and the same 3‐h threshold was therefore used at both sites.

### 2.6 Wintering HRs

Space use by curlews was studied according to three wintering stages with contrasting implications in terms of energy requirements: (1) the first months of the wintering season, i.e., when curlews undergo post‐breeding moult upon arrival at the wintering site and replenish body reserves lost during migration and breeding (Arrival–September; Arr–Sep); (2) mid‐winter, i.e., when the density of birds is relatively stable and the weather is colder (October–January; Oct–Jan); and (3) the last months before departure, i.e., when the curlews undergo pre‐breeding moult and build up body reserves to prepare for migration (February–Departure; Feb–Dep). For each wintering stage, only individuals with ≥ 1000 GPS fixes (Jourdan et al. 2021a) recorded from a minimum of four satellites to increase spatial accuracy (Dutt et al. 2009) were selected for HR analyses (Arr–Sep *n* = 42; Oct–Jan *n* = 44; Feb–Dep *n* = 48) (Table 1). A total of 27 birds were used throughout the three stages (Table 1).

An estimation of the individual utilisation distribution (UD), i.e., the probability density that an animal was present in a given area (Jennrich and Turner 1969), was used to highlight the space used by individuals. UDs were calculated using the kernel density estimation method (KDE; van Winkle 1975) with the ‘kernelUD’ function of the ‘adehabitatHR’ package (Calenge 2023). Individual HRs (95% KDE) and core areas (CAs, 50% KDE) were estimated for each wintering stage (Worton 1989), whatever the birds activity (feeding or resting). The UDs were then calculated based on GPS fixes considered as foraging positions, allowing the estimation of foraging CAs. The cell size was adjusted to 20 m to consider the minimum precision of 10 m of the GPS tags. An 80‐m smoothing was used for all individuals, which is considered as the best compromise between under‐ and over‐smoothing (Donnez et al. 2023). An Albers equal‐area projection adjusted to the extent of wintering GPS fixes was used to project the HRs, allowing the preservation of HR surface areas true to their real area (Snyder 1993) and thus comparing their sizes among all individuals regardless of the study site. All maps were generated using QGIS software (version 3.28 Firenze, QGIS Development Team 2022).

### 2.7 Habitat Selection

The characteristics of the foraging areas selected by curlews throughout the winter were analysed at both sites using resource selection function (RSF) models. The R package ‘amt’ was used to create the random points and to extract the environmental covariates (Signer et al. 2019). Given the different landscapes of the sites, we analysed curlews' habitat selection at the PC and WS using separate models. Landscape layers were first rasterized using a pixel size of 100 m. The habitats were then grouped into six broader habitats before being integrated into the selection models: mudflats, seagrass beds, oyster parks (PC), biogenic reefs (PC), natural habitats (i.e., natural grassland, saltmarsh, marsh and wetland), and agricultural habitats (i.e., arable land and agricultural grassland). For each site, the habitats included in a 99% minimum convex polygon (MCP) of all tagged curlews were considered as available (0) and GPS fixes as used (1) (Thomas and Taylor 1990). For each GPS fix, 10 random fixes were produced within the 99% MCP (Muff et al. 2020). Subtidal areas, dwellings and forests were removed from the MCPs because they were not considered to be available for curlews. Given the vast expanse of the WS considered, the availability of habitats for curlews was carried out according to three districts (i.e., Nordfriesland, Dithmarschen, Cuxhaven and Wesermarsh, see Figure 1), and habitat availability therefore differed depending on the district in which they wintered. Totals of 32 curlews in the PC and 24 in the WS were finally retained for habitat selection analyses, while one curlew that moved between several districts in the WS was excluded.

To determine if individual curlews foraged preferentially far from the coast and close to protected areas, given their sensitivity to human disturbance (Smit and Visser 1993), the effects of distance to the coast and protected areas were included in the selection models.

### 2.8 Statistical Analyses

Most statistical analyses were performed using R software (version 4.4.1, R Core Team 2024). Mean (±SD) wintering duration, arrival and departure dates and number of habitats used were compared by site by Student's *t*‐tests using the ‘*t*.test’ function (‘stats’ package, R Core Team 2024). For non‐normally distributed data, medians were compared by Wilcoxon's rank sum tests using the ‘wilcox.test’ function.

The effects of wintering site (Donnez et al. 2023), sex (Mander et al. 2022), diurnal cycle (day/night) (Jourdan et al. 2021b), and wintering stage on individual HR size (continuous response variable in hectares) were tested jointly using generalised additive mixed models (GAMMs) with the ‘bam’ function (‘mgcv’ package, Wood 2017). A Tweedie distribution was considered to be the most appropriate distribution after comparing the Akaike information criterion (AIC) values for different distribution families and was therefore used to fit the model. Some individuals were used to define multiple HR sizes according to the stage and diurnal cycle, and bird ID and year were therefore defined as random effects. The percentages of HR overlap between day and night and according to wintering stage were calculated using the ‘Overlap Analysis’ tool with QGIS software (QGIS Development Team 2022).

The rhythm of feeding and resting activities of curlews wintering at both sites was estimated. To this end, two GAMMs were performed to compare the proportions of GPS fixes in intertidal areas, except saltmarshes (i.e., potential feeding areas), or on the mainland and in saltmarshes (i.e., potential resting areas) according to the wintering site, wintering stage and tidal cycle. Gaussian and Tweedie distributions, respectively, were used to fit the models after comparing the AIC values, and the bird ID and year were still defined as random effects.

RSF models were fitted by weighted (*W* = 1000) logistic regression models using the ‘glmmTMB’ R package (McGillycuddy et al. 2025), with both individual‐specific random intercepts and coefficients (Muff et al. 2020). Continuous predictors were centred and scaled to facilitate comparison between estimates.

## 3 Results

### 3.1 Comparison Between Sites

#### 3.1.1 HR Sizes

**FIGURE 3:**
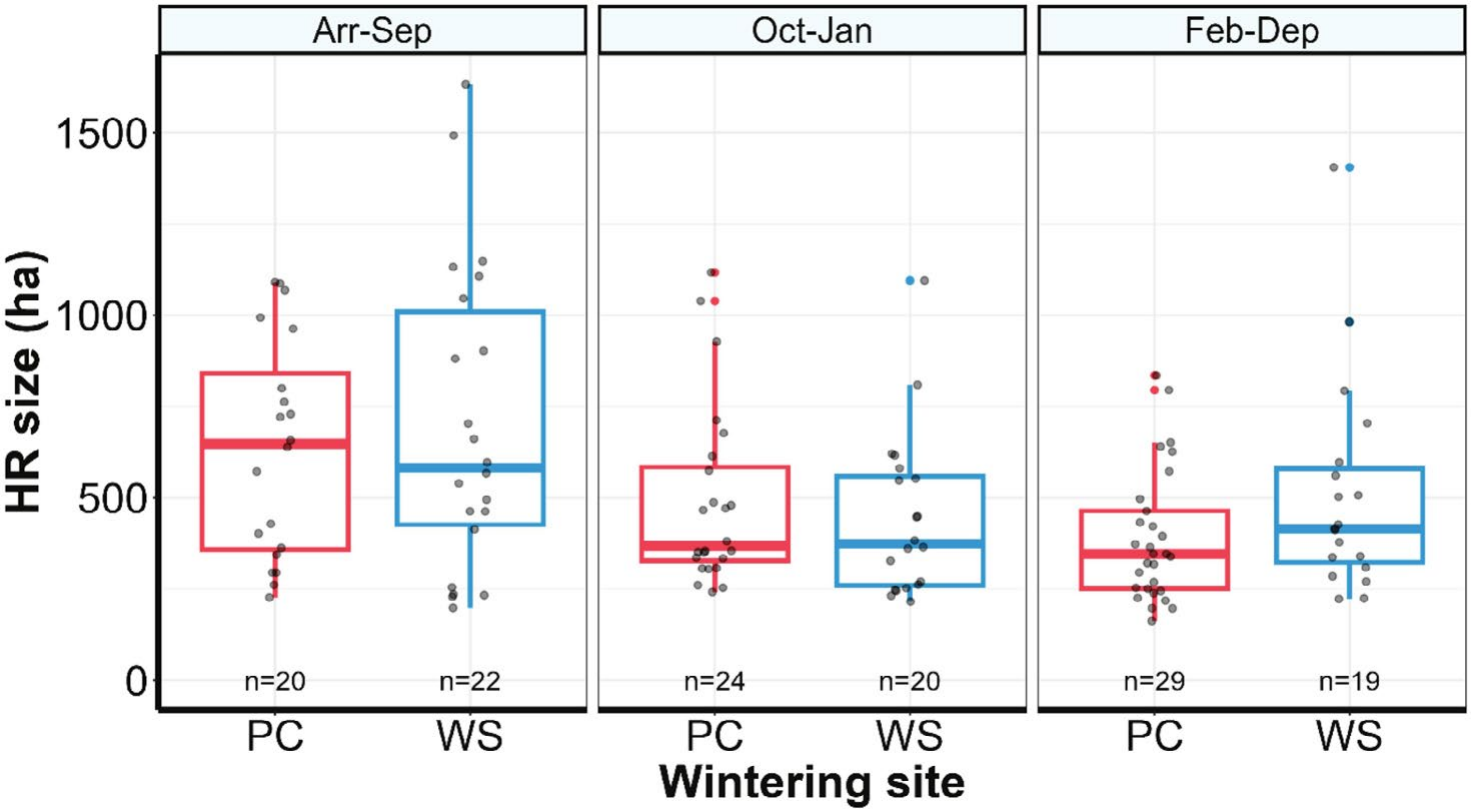
Home range (HR) sizes for curlews wintering in the Pertuis Charentais (PC) in France and in the Wadden Sea (WS) in Germany according to the three wintering stages. Medians and numbers of individuals per category are shown.

Tagged curlews had mean (±SD) global wintering HRs of 726 ± 252 ha (334–1291 ha, *n* = 14) in the PC and 644 ± 339 ha (233–1203 ha, *n* = 13) in the WS. Their average global CAs were 103 ± 40 ha (60–190 ha, *n* = 14) and 98 ± 57 ha (18–204 ha, *n* = 13), respectively. Curlews thus exhibited similar HR sizes at both sites, regardless of the wintering stage (GAMM: coefficient = 0.15, 95% CI = [−0.08, 0.38], *p* = 0.21) (Figure 3, Table 2).

**TABLE 2:** GAMM results showing partial effects of selective explanatory variables on global wintering home range size in wintering curlews (*n* = 62).

Parametric coefficient	Estimate	Std.error	*t* value	*p*
Intercept	6.34	0.11	56.59	***
Site (WS)	0.15	0.12	1.25	0.21
Sex (male)	−0.16	0.11	−1.42	0.16
Diurnal cycle (night)	−0.21	0.04	−5.58	***
Stage (Oct–Jan)	−0.46	0.05	−9.13	***
Stage (Feb–Dep)	−0.46	0.05	−8.92	***

	edf	Ref.df	*F* value	*p*
s(Bird.ID)	45.24	56	8.13	***
s(Year)	3.67	9	13.90	0.20

*Note:* Significance of individual model predictors: ***p < 0.001. Relative proportion of overall variance explained = 70%.

Abbreviations: edf, effective degrees of freedom; Ref.df, reference number of degrees of freedom; Std.error, standard error.

There was no significant difference in global wintering HR size between the sexes (coefficient = −0.16, *p* = 0.16). Curlews exploited larger global HR surfaces during the day (879 ± 491 ha, *n* = 27) than during the night (689 ± 337 ha, coefficient = −0.21, *p* < 0.001) (Table 2), although global day and night HRs overlapped at 76% ± 11%.

#### 3.1.2 Rhythm of Feeding and Resting Activities

**FIGURE 4:**
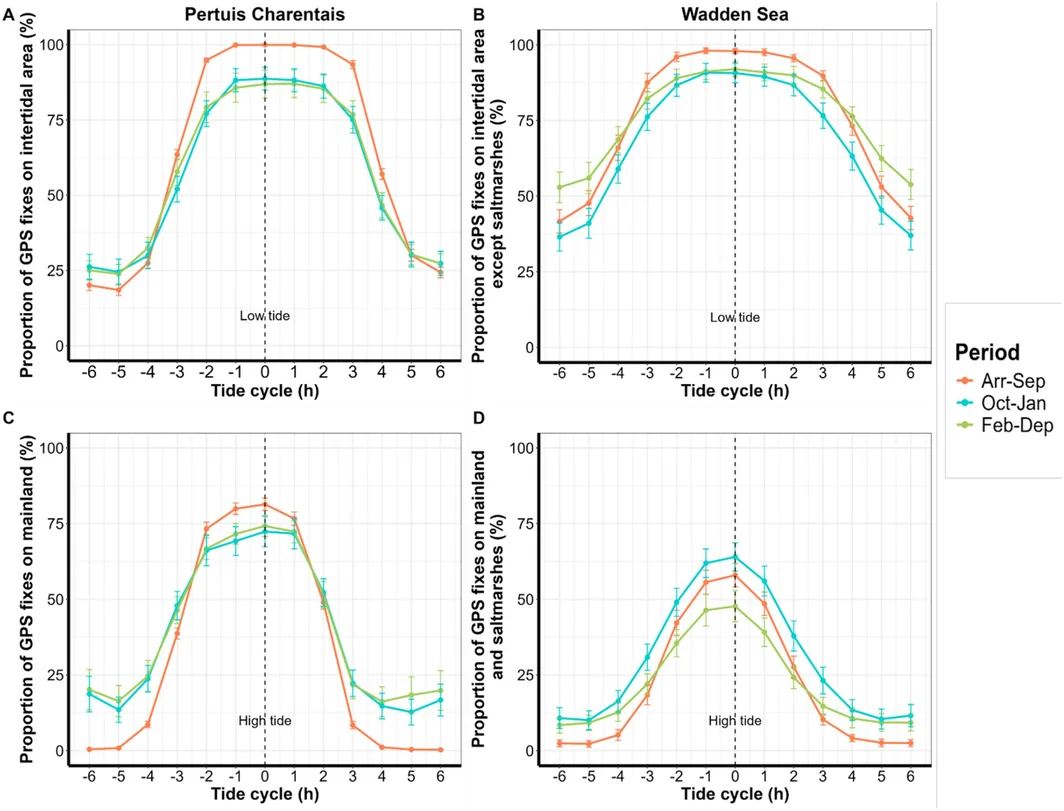
Mean (± standard error) proportion of time spent by wintering curlews on intertidal areas according to low tide time (A, B) and on mainland or in saltmarshes according to high tide time (C, D) during the tidal cycle in the Pertuis Charentais (left column) and the Wadden Sea (right column).

The GPS fixes located in intertidal areas (except saltmarshes), i.e., in potential feeding areas, indicated that curlews foraged during approximately 5–7 h by tide cycle in the PC and 6–8 h in the WS. The presence in intertidal areas was greater from about 1 h before to 2 h after low tide (GAMM: *p* ≥ 0.38, with *p* the probability that the observed presence of curlews in intertidal areas every hour of offset with low tide do not differ from the presence of curlew at low tide), suggesting higher feeding activity (Figure 4A,B and Table A1). Curlews wintering in the WS thus spent more time on the mudflats than curlews wintering in the PC, particularly around high tide (*p* < 0.001).

According to the GPS fixes located on the mainland and in saltmarshes, i.e., potential resting areas, curlews seemed to rest mainly from about 2 h before and 1 h after high tide (GAMM: *p* ≥ 0.20) (Figure 4C,D and Table A1). Curlews were also likely to rest on the mudflats when the water level allowed, especially at the upper part of the intertidal area. Curlews from the WS seemed to have more opportunities to rest on intertidal areas, with a lower proportion of GPS fixes on potential mainland roosts (*p* < 0.001).

#### 3.1.3 Foraging Habitat Use

**FIGURE 5:**
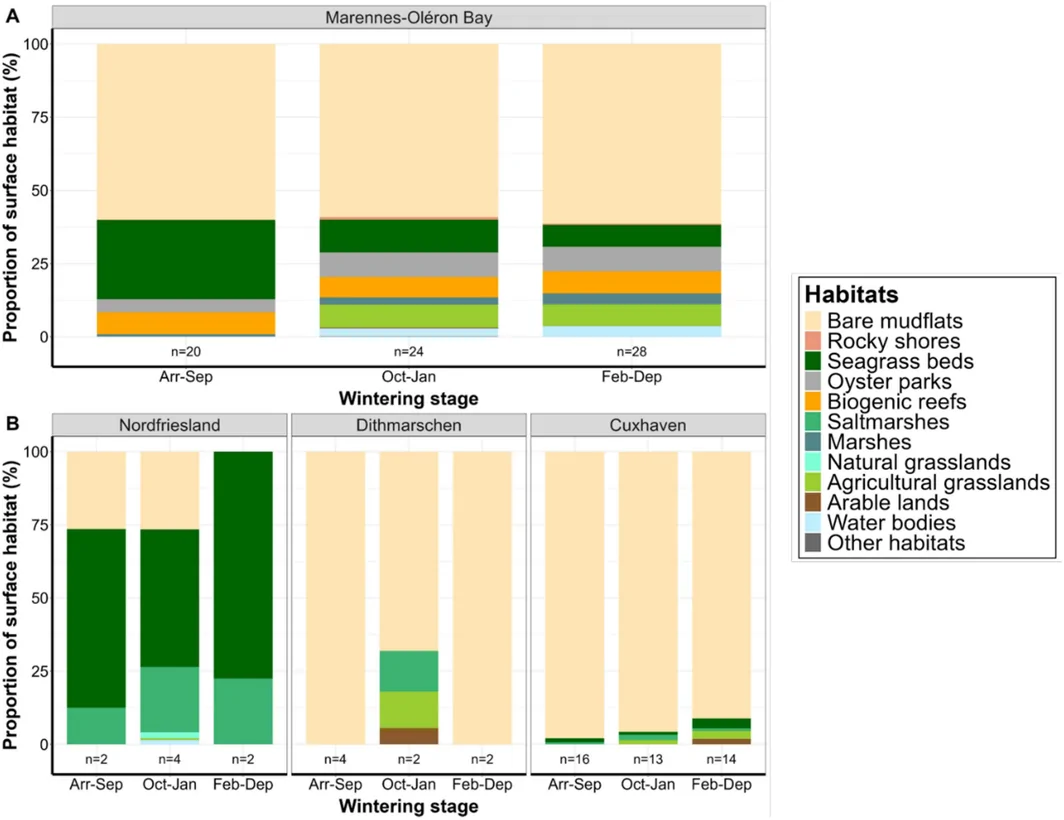
Proportion of habitat use within the foraging core area according to wintering stage in the Pertuis Charentais (A) and Wadden Sea (B).

PC curlews used more habitats than individuals wintering in the WS (mean ± SD (range): 7.4 ± 2.0 (4–11) vs. 4.9 ± 1.6 (2–8) habitats) when accounting for foraging HR surfaces (Student's *t*‐test, *t* = 5.29, *p* < 0.001). Similarly, wintering curlews used totals of 4.5 ± 2.2 (1–10) habitats and 2.5 ± 1.7 (1–7) habitats, respectively, when accounting for foraging CA surfaces (Figure 5). Curlews in the WS fed mainly on the mudflats or seagrass beds in case of curlews from Nordfriesland, while curlews in the PC fed on the mudflats, but also on biogenic reefs, oyster parks and seagrass beds.

### 3.2 Comparison Between Winter Stages

#### 3.2.1 HR Sizes

**FIGURE 6:**
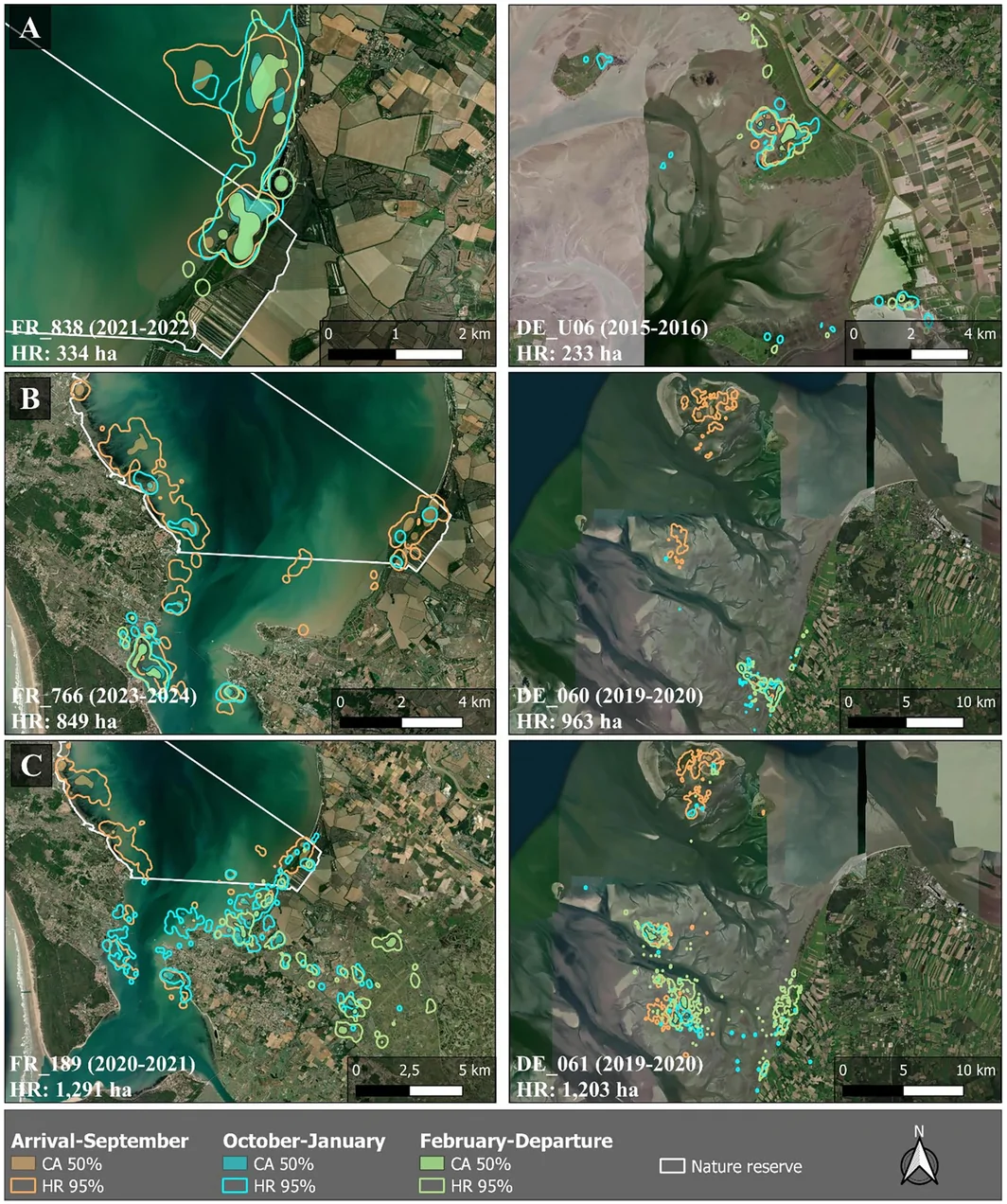
Three periodic home ranges (HRs) (95%) and core areas (CAs) (50%) for curlews wintering in the Pertuis Charentais (PC) (left column) and Wadden Sea (WS) (right column), from the smallest (A) and intermediate (B) to the largest (C) global HR. Individual code number, wintering season and global HR size in ha are shown.

Curlews consistently showed significantly larger HRs in both study areas during the first wintering stage (Arr–Sep: 669 ± 362 ha) compared with the second (Oct–Jan: 468 ± 235 ha, *t* = −9.10, *p* < 0.001) and third wintering stages (Feb–Dep: 437 ± 237 ha, *t* = −8.93, *p* < 0.001) (Figures 3 and 6, Table 2). Moreover, there was less HR overlap between the first and second stages (42% ± 19%, *n* = 26) than between the second and third stages (63% ± 18%, *n* = 26; paired *t*‐test: *t* = −5.75, *p* < 0.001), indicating different individual‐space use at the beginning of the wintering season (Figure 6).

#### 3.2.2 Foraging Habitat Use

**FIGURE A2:**
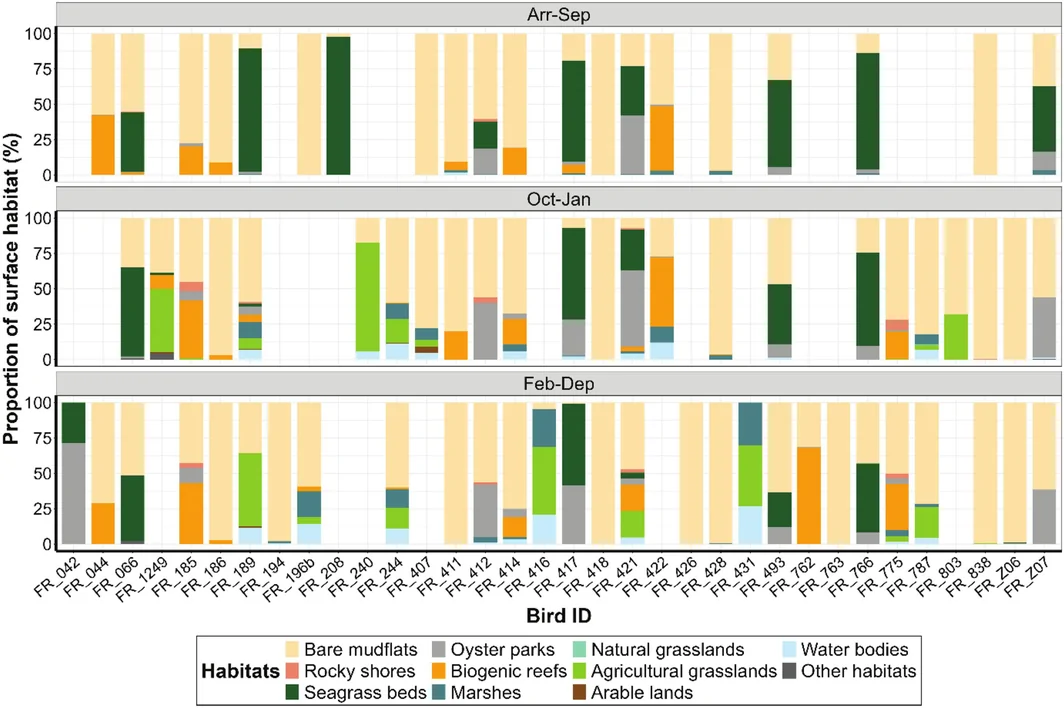
Proportion of habitat use within the foraging core area for each individual according to the wintering stage in the Pertuis Charentais.

**FIGURE A3:**
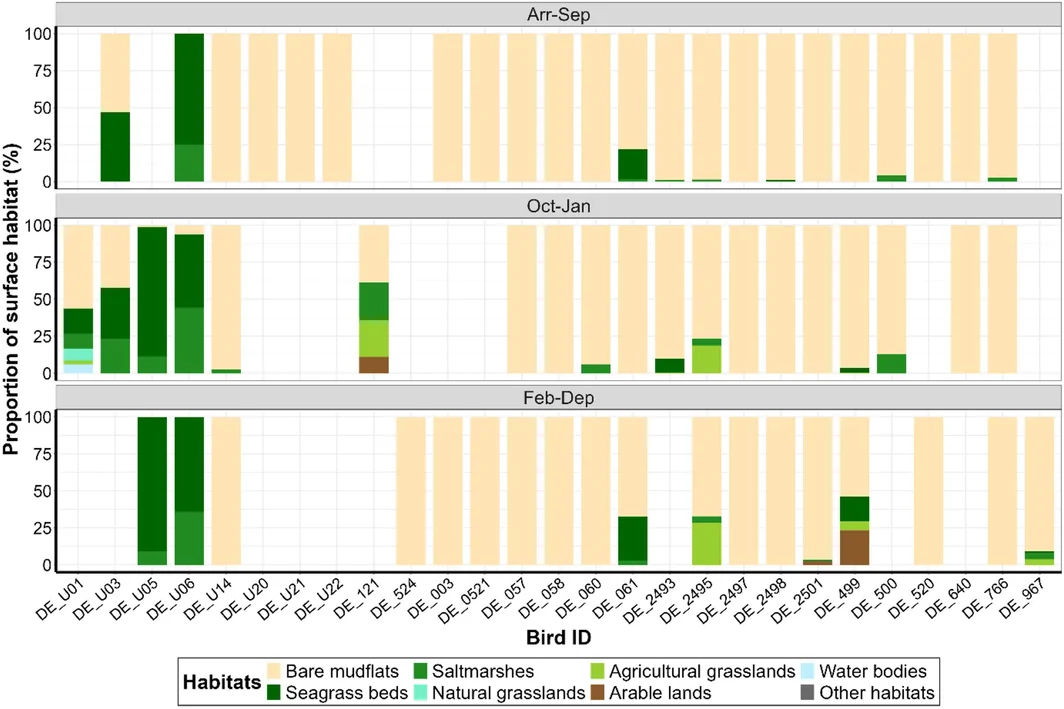
Proportion of habitat use within the foraging core area for each individual according to the wintering stage in the Wadden Sea.

On average, curlews stayed on the mudflat more from their arrival to September, compared with the other two stages, irrespective of the site (GAMM, *p* < 0.001, Figure 4). Curlews at both sites almost exclusively used the intertidal areas during the first stage (Arr–Sep), especially bare mudflats (PC: 60% of surface habitat; WS: 92% of surface habitat), except for curlews wintering on the coast of Nordfriesland where they more often frequented seagrass beds (61%) and saltmarshes (13%) compared with other German tagged curlews. Seventeen curlews of both sexes in the PC and twelve in the WS started to use inland habitats during the second (Oct–Jan) and third (Feb–Dep) stages. In particular, agricultural grasslands accounted for 7%–8% of the total surface habitat of a foraging CA during these two stages in the PC and 2% in the WS (Figure 5 and Figures A2 and A3).

#### 3.2.3 Foraging Habitat Selection

Given that space use was the same in the second and the third stages, only habitats selected during the first stage (Arr–Sep) were compared with those selected during the last stage (Feb–Dep). The probability of a foraging curlew being present decreased in relation to distance from the coast at both sites (RSF, *p* < 0.01 in PC and *p* < 0.001 in WS) (Table 3), although curlews foraged further from the coast during the first winter stage, foraging closer to protected areas in PC, compared with the third stage (*p* < 0.001). As expected, mudflats were preferred by curlews for foraging compared with inland habitats (i.e., agricultural and natural habitats) (*p* < 0.001), especially in the WS, where mudflats were frequently the only habitat used by many birds (*p* < 0.001). Biogenic reefs were also highly selected in the PC, regarding their low availability. Curlews from the PC demonstrated higher selection of intertidal habitats during the first stage, especially seagrass beds, compared with the third stage (*p* < 0.001). In contrast, curlews seemed to select mudflats and seagrass beds more during this first stage in the WS, although the difference with the third stage was less pronounced (*p* < 0.001). Natural habitats, i.e., primarily saltmarshes in the WS and marshes in the PC, were also selected more often by curlews during this first stage (*p* < 0.001).

**TABLE 3:** Estimated model coefficients for resource selection function models of factors affecting foraging‐area selection by wintering curlews in the Pertuis Charentais (PC) and Wadden Sea (WS).

Predictor	PC			WS		
	Odds ratio	95% CI	*p*	Odds ratio	95% CI	*p*
Intercept	0.00	0.00–0.00	***	0.00	0.00–0.00	***
Habitat (mudflat)	73.49	22.81–236.84	***	182.22	63.76–520.76	***
Habitat (seagrass beds)	0.03	0.00–1.02	0.05	9.47	1.80–49.77	**
Habitat (oyster parks)	0.31	0.02–6.18	0.44	n.c.	n.c.	n.c.
Habitat (biogenic reefs)	21.22	4.07–110.55	***	n.c.	n.c.	n.c.
Habitat (natural)	11.45	5.39–24.33	***	28.91	7.98–104.67	***
Stage (Arr–Sep)	0.00	0.00–0.01	***	0.14	0.12–0.15	***
Distance to mainland (m)	0.54	0.46–0.64	**	0.02	0.00–0.06	***
Distance to protected area (m)	0.00	0.00–0.05	***	0.03	0.00–7.26	0.21
Mudflat: Arr–Sep	114.57	81.17–161.70	***	8.66	7.69–9.76	***
Seagrass beds: Arr–Sep	342.25	242.24–483.55	***	15.67	13.78–17.82	***
Oyster parks: Arr–Sep	67.71	47.86–95.79	***	n.c.	n.c.	n.c.
Biogenic reefs: Arr–Sep	45.43	32.13–64.23	***	n.c.	n.c.	n.c.
Natural: Arr–Sep	135.82	96.05–192.06	***	28.68	23.50–30.29	***
Distance to mainland: Arr–Sep	1.68	1.66–1.69	***	3.34	3.27–3.43	***
Protected areas: Arr–Sep	0.61	0.60–0.62	***	1.16	1.14–1.18	***

*Note:* Habitat selection was compared between the third and first stages (Arr–Sep). Significance of individual model predictors: **p < 0.01, ***p < 0.001.

Abbreviations: CI, confidence interval; n.c., not concerned.

## 4 Discussion

### 4.1 Comparison Between Sites

#### 4.1.1 Similar HR Sizes

Curlews exhibited similar HR sizes at the PC and WS, independent of the winter stage and despite different types of harness and material used to track the birds. This was unexpected, given the higher overall density of birds in the WS that could force birds to cover more space to reduce competition for resources (Leone and Estévez 2008). Indeed, a previous study comparing the HR sizes of curlews from 66 different wintering sites showed that curlews in the WS exhibited larger HRs (mean ± SD: 902 ± 523 ha in WS vs. 466 ± 355 ha in the PC), without identifying any reliable explanatory variable (Donnez et al. 2023). The apparently contradictory results of these two studies are thus surprising, given that both used the same methods and some of the same individuals. Notably, however, the first study included other curlews wintering in the Dutch WS and in Ré Island in the PC, and it is therefore possible that the observed difference was only related to these individuals, and that the space use of curlews from the German WS is not affected by interference and prey depletion related to strong intra‐ or inter‐specific competition. However, curlews from WS spent more time on mudflat; we can therefore not exclude that they extended their temporal foraging window to compensate for the greater competition.

The HR sizes were highly variable among individuals from the same site, not related to their sex. Previous studies also found no sex effect on HR sizes of wintering curlews (Mander et al. 2022; Donnez et al. 2023), even though males and females could have distinct foraging behaviours during winter. Indeed, a preponderance of males in flocks feeding on pastures has been noted at several sites (Ens 1983; Pell et al. 2023), while females were more likely to stay on the mudflats (Townshend 1981). In the current study, however, both males and females used agricultural grasslands, with no observed preference for either sex.

Curlews at both sites exhibited larger HRs during the day than at night, which was not related to distinct use of foraging habitats, as reported for bar‐tailed godwits *Limosa lapponica* (Jourdan et al. 2021b). Indeed, the high overlap between day and night HRs confirmed the high fidelity of curlews to their feeding territories at night (Cramp and Simmons 1983; Mcneil et al. 1992). As a result, the smaller HRs at night may reflect less human disturbance, e.g., within oyster parks, which could lead to the use of significant amounts of time and energy for escape flights (Goss‐Custard et al. 2006), fewer feeding opportunities (de Boer and Longamane 1996; Navedo and Masero 2007), and avoidance of some foraging areas (Navedo and Herrera 2012). Human disturbance, however, is limited on WS mudflats due to strong protection schemes. Furthermore, curlews may also avoid nearshore feeding and roosting areas because of a greater risk of avian predation (Piersma et al. 2006), potentially reducing their night HRs. Conversely, Mander et al. (2022) showed that curlews wintering in the Humber Estuary (UK) exhibited greater nocturnal than diurnal ranges, but with high variability among individuals.

#### 4.1.2 Contrasting Activity Rhythms

Although the HR sizes were comparable between sites, curlews exhibited different feeding and resting activity rhythms, with longer opportunities to feed on mudflats in the WS. Indeed, curlews in the WS fed further from the coast and could enter the mudflats earlier than curlews in the PC. The WS site exhibits high morphological dynamics, with several shoals (Wang et al. 2012) that can be used as intertidal roosts, while curlews in the PC systematically switched between using intertidal feeding areas around low tide and supratidal (mainly mainland) resting areas around high tide. Time on the mudflats was not symmetrical for PC curlews, with the highest feeding activity from 1 h before to 2 h after low tide. The same pattern was observed in bar‐tailed godwits *Limosa lapponica* wintering in Re Island (Jourdan et al. 2021a). In contrast, the proportions of foraging curlews in the Humber estuary were similar 3 h either side of low tide (median: 80%–100%; Mander et al. 2022), resembling the pattern found in the WS. Hence, the feeding activity rhythm was different between sites, despite the same 3‐h threshold. It should be noted that an Expectation Maximisation binary Clustering (EMbC) algorithm based on the speed and turning angle between successive locations was primarily tested to better differentiate individual behaviours (Mendez et al. 2017), but the definition of feeding and resting activities failed.

#### 4.1.3 Distinct Foraging‐Habitat Availability

As expected, curlews fed mainly on intertidal mudflats where they can find their preferential prey items, i.e., polychaete worms, bivalves and crustaceans (Goss‐Custard et al. 1977; Bocher et al. 2014, A. Bange, *pers. obs*.). Overall, individuals in the PC exploited several intertidal habitats, while curlews in the WS essentially used the mudflats for feeding. Indeed, the WS landscape offered fewer possibilities in terms of cover types compared with the PC site. However, the separation of foraging and resting positions was based on the behaviour of PC curlews; it is therefore possible that the real proportion of foraging habitat use in WS is slightly different, suggesting that WS curlews continue to feed outside this 6‐h foraging window. Several curlews in the PC used biogenic reefs resulting from former and/or current oyster parks for feeding. The substrate under the oyster beds is rich in organic carbon due to the deposition of faeces from the oysters, favouring the presence of polychaetes (Sornin et al. 1983; Castel et al. 1989; Jourdan et al. 2021b). Surprisingly, a high abundance of curlews also foraged on seagrass beds, especially in the northern WS, even though previous studies indicated their minor importance as a food source for non‐herbivorous birds wintering in the Sylt‐Rømø Bight (Busch 2012). Horn et al. (2017) attributed this unexpected foraging behaviour in the WS to a long exposure time of seagrass meadows and to less human disturbance compared with other bays, where seagrass meadows often occur close to the shore; however, these assumptions were not supported by the observations in the PC.

### 4.2 Comparison Between Winter Stages

#### 4.2.1 Feeding Areas Shifting

Tagged curlews almost exclusively used the intertidal area to feed at the beginning of the wintering season compared with later in the season; however, it is unclear whether this pattern was due to higher feeding activity or because some curlews started foraging on farmland in mid‐winter and therefore spent less time on the mudflat. Clearly, however, curlews were more mobile during this first stage, as suggested by their larger HRs. Given the high interannual overlap between their HRs in these sites (unpubl. data), it is unlikely that curlews need to explore the sites in search of suitable new feeding areas. This pattern is more likely to be related to a higher energy demand at the beginning of the wintering season due to previous long‐distance migration and moult, assuming that higher energy requirements result in greater spatial use (McNab 1963; Tamburello et al. 2015).

Like many shorebirds (see Higgins and Davies 1996), curlews often conduct most or all of their primary moult at their wintering grounds (approx. 81–112 days from July to December; Géroudet 1983). Their flight ability is impaired during moult, and shorebirds are thus more‐exposed to predation risks due to their reduced escape flight performance (Kjellén 1994; Jackson and Underhill 2022). This could explain why curlews fed further from the coast at this time (as demonstrated by curlews frequenting the remote WS islands of Scharhörn and Nigehörn). Marks et al. (1990) previously found that bristle‐thighed curlews *Numenius tahitiensis* wintered on remote Hawaiian Islands free of predators because their flight ability was compromised by their primary moult. This could also explain the higher use of protected areas during this first stage. Indeed, Jiguet et al. (2023) showed that adult curlews benefitted from the experience of previous wintering seasons to forage primarily inside the nature reserve during the daytime in the PC, to overcome human disturbances (e.g., hunting). Energy demands, however, cannot be the only reason for the larger HRs, otherwise the HRs would also have been larger prior to migration when fuelling is needed and the energy demand is high (Wiersma and Piersma 1994).

#### 4.2.2 Contrasting Foraging Habitat Selection

Similar to the size and location of HRs, the foraging habitats varied between the first and the other two winter stages, potentially reflecting a different prey supply. Indeed, birds have more need for protein during moulting to supply substrate and for energy (Murphy and King 1992; McWilliams 2008). The curlew is the first species to reach their wintering site after the autumn migration (P. Rousseau, Manager of the Moëze‐Oléron Nature Reserve, *pers. obs*.), thus reducing inter‐specific competition for food resources and allowing them to explore sites in search of high‐quality foraging areas that they may not be able to frequent afterwards. As a result, curlews exploited the seagrass beds in the PC more during the first wintering stage, i.e., when it was not being used by other shorebirds and geese. Globally, seagrass beds are rich in benthic macrofauna due to the organic detritus they provide, including many polychaetes (Castel et al. 1989) and shore crabs ( *Carcinus maenas* ) in summer (Heck et al. 1995), which are particularly rich in protein (Zwarts and Blomert 1990; Naczk et al. 2004). Annelids are more difficult to probe in this habitat, and curlews are more likely to use it because of the high abundance of crabs, which can be pecked from the surface. Indeed, Navedo and Masero (2008) showed that an intertidal area almost totally covered by seagrass in the Santoña Marshes Natural Park (Spain) supported a high density of curlews during post‐breeding migration (July–September), feeding on shore crabs (Navedo and Masero 2007). Schwemmer et al. (2012) accordingly found a relatively high frequency (28.6%) of shore crabs in the stomachs of seven curlews collected in summer and autumn in the German WS.

Several individuals supplemented their food resources within terrestrial habitats during the course of the winter, especially on agricultural grasslands. Curlews could switch to inland foraging habitats when the availability of polychaetes and some bivalve species in intertidal habitats declined, in line with decreasing local temperatures and day length (Townshend 1981; Zwarts and Wanink 1993). Inland foraging freed individuals from the tide schedule (Gutiérrez et al. 2012), allowing them to feed on earthworms throughout the day (Navedo et al. 2020). Moreover, by foraging on soft‐bodied prey items, shorebirds avoid the digestive bottlenecks generated by large and hard‐shelled prey items such as crustaceans (van Gils et al. 2005; Navedo et al. 2013). Indeed, Zharikov and Skilleter (2003) showed that wintering Far‐eastern curlews feeding mainly on crabs were unable to increase their intake rate or digestive efficiency before long‐distance migration because of this digestive constraint. Selecting different habitats during the course of the winter may thus allow curlews to adopt different strategies to deal with their energy demands throughout the wintering season.

## 5 Conclusions

This study highlights the similar wintering strategies of curlews in terms of space use between two major wintering sites in Europe, with longer use of mudflats in the WS and more foraging habitat availability in the PC. The results also demonstrate the different habitat‐selection strategies at both sites between the first months of the wintering season and later stages. Contrary to our initial hypothesis, curlews exhibited similar HR size at both sites, suggesting that the greater density of waterbirds in the WS has no effect on individuals' space use. Nevertheless, we cannot exclude the possibility that curlews in the WS were forced to stay longer on the mudflats compared with curlews in the PC in order to satisfy their energy demands, especially due to the more energetically costly conditions in the WS (Wiersma and Piersma 1994); however, only a few individuals made use of terrestrial habitats to gain more prey during high tide. Overall, curlews used more intertidal areas at the beginning of the season, probably due to their higher energy requirements during this stage as well as to weaker inter‐specific competition. There was no notable change in spatial behaviour between mid‐winter and late winter, even though migratory fuel is considered to account for 10%–13% of curlews' body mass in April (Summers et al. 2023). Finally, this study demonstrated that curlews from two wintering sites with distinct characteristics, but sharing the same breeding areas in North‐Eastern Europe, seem to exhibit similar wintering strategies prior to the upcoming breeding season. A greater consideration of the distribution of curlews and other shorebirds at the beginning of the wintering season is, moreover, recommended to identify a possible link to changing patterns of invertebrates.

## Author Contributions

**Marie Donnez:** conceptualization (equal), data curation (equal), formal analysis (equal), investigation (equal), methodology (equal), software (lead), validation (equal), visualization (equal), writing – original draft (lead), writing – review and editing (lead). **Philipp Schwemmer:** conceptualization (equal), data curation (equal), formal analysis (equal), funding acquisition (equal), investigation (equal), methodology (equal), project administration (equal), resources (equal), supervision (equal), validation (equal), visualization (equal), writing – original draft (equal), writing – review and editing (equal). **Moritz Mercker:** formal analysis (supporting), software (supporting), writing – original draft (supporting). **Stefan Garthe:** data curation (equal), funding acquisition (equal), resources (supporting), writing – original draft (supporting). **Frédéric Jiguet:** data curation (equal), funding acquisition (equal), resources (equal), writing – original draft (supporting). **Andreas Bange:** investigation (supporting), writing – original draft (supporting). **Loïc Jomat:** data curation (equal), funding acquisition (supporting), investigation (supporting), resources (supporting), writing – original draft (supporting). **Pierre Rousseau:** data curation (equal), funding acquisition (supporting), investigation (supporting), resources (supporting), writing – original draft (supporting). **Pierrick Bocher:** conceptualization (equal), data curation (equal), formal analysis (equal), funding acquisition (equal), investigation (equal), methodology (equal), project administration (equal), resources (equal), supervision (equal), validation (equal), visualization (equal), writing – original draft (equal), writing – review and editing (equal).

## Funding

The capture, ringing, and tagging of curlews in France were licenced by the CRBPO (French national ringing scheme) under reference number PP1083 and funding for work was provided as part of the ECONAT project funded by the Contrat de Plan Etat‐Région, the CNRS and the European Regional Development Fund (QUALIDRIS project), and by the Ligue pour la Protection des Oiseaux. Permission to attach GPS tags in Germany was issued by the Ministerium für Energiewende, Landwirschaft, Umwelt, Natur und Digitalisierung of the federal state of Schleswig‐Holstein (file numbers V 312‐7224.121‐37(42‐3/13) and V 241‐35852/2017(88‐7/17)) as well as by the Niedersächsisches Landesamt für Verbraucherschutz und Lebensmittelsicherheit of the federal state of Lower Saxony (file number 33‐19‐42502‐04‐17/2699). Parts of this study were conducted in Germany by the projects ‘Birdmove’ (FKZ 3515822100) and ‘Trackbird’ (FKZ 3519861400) funded by the Federal Agency for Nature Conservation as well as the project ‘Triple crisis meets trilateral cooperation: Effects of biodiversity loss, climate change and pollution on salt marshes & pathways to their sustainable management’ of the research programme ‘Understanding complex pressures on the Wadden Sea and options for action’, which is financed by the Dutch Research Council (NWO), the German Federal Ministry of Research, Technology and Space (BMFTR) and the Federal Ministry for the Environment, Nature Conservation and Nuclear Safety (BMUKN) under the grant numbers NWA.1438.20.005 and FKZ 03F0960 C. This study is included in the HABITRACK project funded by the European Union through their HORIZON Research and Innovation Actions (Project 101135047, 659 HORIZON‐CL6‐2023‐BIODIV‐01‐Biodiversity and Ecosystem Services). Views and opinions expressed are however those of the authors only and do not necessarily reflect those of the European Union or the European Research Executive Agency (REA). Neither the European Union nor the granting authority can be held responsible for them.

## Conflicts of Interest

The authors declare no conflicts of interest.

## Data Availability

Data is available from the Dryad Digital Repository: https://doi.org/10.5061/dryad.nvx0k6f6j (Donnez et al. 2026).

## Appendix A

**TABLE A1:** GAMM results showing partial effects of selective explanatory variables on the proportion of GPS fixes for wintering curlews on mudflats and on the mainland and saltmarshes (*n* = 60).

Parametric coefficient	Mudflat				Mainland			
	Estimate	Std.error	*t* value	*p*	Estimate	Std.error	*t* value	*p*
Intercept	92.72	5.22	17.76	***	4.15	0.32	13.08	***
Site (WS)	1.79	4.45	0.40	0.69	−4.29	0.31	−13.79	***
Stage (Oct–Jan)	−4.43	1.04	−4.27	***	0.12	0.06	1.91	0.06
Stage (Feb–Dep)	−7.73	1.07	−7.25	***	0.37	0.07	5.67	***
Tide (−6)	−67.00	1.83	−36.70	***	−2.24	0.13	−16.74	***
Tide (−5)	−68.48	1.83	−37.41	***	−2.54	0.11	−22.98	***
Tide (−4)	−60.80	1.83	−33.30	***	−1.66	0.10	−16.55	***
Tide (−3)	−33.61	1.83	−18.41	***	−0.62	0.09	−6.65	***
Tide (−2)	−8.28	1.83	−4.54	***	−0.12	0.09	−1.30	0.20
Tide (−1)	0.65	1.83	−0.36	0.72	−0.03	0.09	−0.38	0.70
Tide (+1)	−0.13	1.83	−0.07	0.94	−0.04	0.09	−0.41	0.68
Tide (+2)	−1.62	1.83	−0.88	0.38	−0.46	0.09	−4.96	***
Tide (+3)	−10.30	1.83	−5.64	***	−1.75	0.10	−17.37	***
Tide (+4)	−41.87	1.83	−22.94	***	−2.49	0.11	−23.01	***
Tide (+5)	−60.86	1.83	−33.34	***	−2.59	0.12	−22.14	***
Tide (+6)	−64.56	1.83	−35.37	***	−2.28	0.13	−17.43	***
Stage (Oct–Jan): Site (WS)	0.56	1.46	0.38	0.70	1.51	0.14	11.00	***
Stage (Feb–Dep): Site (WS)	6.37	1.51	4.21	***	1.84	0.13	13.87	***
Tide (−6): Site (WS)	16.60	2.72	6.11	***	1.35	0.23	5.87	***
Tide (−5): Site (WS)	22.73	2.72	8.36	***	1.72	0.22	7.99	***
Tide (−4): Site (WS)	31.52	2.72	11.60	***	0.82	0.21	4.02	***
Tide (−3): Site (WS)	22.05	2.72	8.11	***	0.06	0.19	0.34	0.74
Tide (−2): Site (WS)	5.33	2.72	1.96	0.50	−0.20	0.19	−1.06	0.29
Tide (−1): Site (WS)	0.51	2.72	0.19	0.85	−0.06	0.18	−0.33	0.74
Tide (+1): Site (WS)	−0.73	2.72	−0.27	0.79	−0.00	0.18	−0.02	0.99
Tide (+2): Site (WS)	−1.18	2.72	−0.43	0.66	0.14	0.19	0.79	0.43
Tide (+3): Site (WS)	0.63	2.72	0.23	0.82	1.20	0.20	6.07	***
Tide (+4): Site (WS)	18.98	2.72	6.98	***	1.64	0.21	7.94	***
Tide (+5): Site (WS)	20.45	2.72	7.52	***	1.69	0.22	7.79	***
Tide (L6): Site (WS)	15.01	2.72	5.52	***	1.34	0.23	5.83	***

	edf	Ref.df	*F* value	*p*	edf	Ref.df	*F* value	*p*
s(Bird.ID)	53.07	60	187.5	0.08	52.9	60	210.3	**
s(Year)	7.57	9	3933.9	***	7.18	9	1820.5	*

*Note:* Significance of individual model predictors: **p* < 0.05, ***p* < 0.01, ****p* < 0.001.
